# (R*)-Methyl 2-(2,6-dimeth­oxy-3,5-di­nitro­benzamido)­propano­ate

**DOI:** 10.1107/S1600536812029935

**Published:** 2012-07-07

**Authors:** Xiaofei Li, Yan Tong

**Affiliations:** a College of Pharmacy, Henan University of Traditional Chinese Medicine, Zhengzhou 450008, People’s Republic of China

## Abstract

In the title mol­ecule, C_13_H_15_N_3_O_9_, the nitro groups are tilted with respect to the benzene mean plane by 22.8 (3) and 31.6 (3)°. The meth­oxy groups are in a *cis* orientation relative to the ring. In the crystal, mol­ecules are linked by strong N—H⋯O hydrogen bonds into *C*(3) chains along [100].

## Related literature

For the biological activity of related compounds or for their use as prodrugs, see: Sykes *et al.* (1999 ▶). For hydrogen-bond motifs, see: Bernstein *et al.* (1995 ▶).
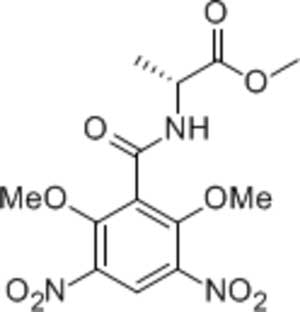


## Experimental

### 

#### Crystal data

C_13_H_15_N_3_O_9_
*M* *_r_* = 357.28
Orthorhombic, 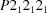

*a* = 4.6933 (10) Å
*b* = 17.501 (3) Å
*c* = 19.917 (4) Å
*V* = 1635.9 (6) Å^3^
*Z* = 4
Mo *K*α radiation
μ = 0.13 mm^−1^
*T* = 296 K
0.31 × 0.30 × 0.09 mm

#### Data collection

Bruker APEXII CCD area-detector diffractometer
Absorption correction: multi-scan (*SADABS*; Sheldrick, 1996 ▶) *T*_min_ = 0.962, *T*_max_ = 0.989
9526 measured reflections
3683 independent reflections
2824 reflections with *I* > 2σ(*I*)
*R*_int_ = 0.025

#### Refinement

*R*[*F* ^2^ > 2σ(*F* ^2^)] = 0.045
*wR*(*F* ^2^) = 0.153
*S* = 1.03
3683 reflections
226 parameters
H-atom parameters constrained
Δρ_max_ = 0.22 e Å^−3^
Δρ_min_ = −0.17 e Å^−3^

### 

Data collection: *APEX2* (Bruker, 2007 ▶); cell refinement: *SAINT* (Bruker, 2007 ▶); data reduction: *SAINT*; program(s) used to solve structure: *SHELXTL* (Sheldrick, 2008 ▶); program(s) used to refine structure: *SHELXTL*; molecular graphics: *SHELXTL*; software used to prepare material for publication: *SHELXTL* and *publCIF* (Westrip, 2010 ▶).

## Supplementary Material

Crystal structure: contains datablock(s) I, global. DOI: 10.1107/S1600536812029935/bx2416sup1.cif

Structure factors: contains datablock(s) I. DOI: 10.1107/S1600536812029935/bx2416Isup2.hkl

Supplementary material file. DOI: 10.1107/S1600536812029935/bx2416Isup3.cml

Additional supplementary materials:  crystallographic information; 3D view; checkCIF report

## Figures and Tables

**Table 1:** Hydrogen-bond geometry (Å, °)

*D*—H⋯*A*	*D*—H	H⋯*A*	*D*⋯*A*	*D*—H⋯*A*
N3—H3*A*⋯O2^i^	0.86	2.02	2.850 (2)	162

Symmetry code: (i) 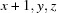.

**Fig. 1.**
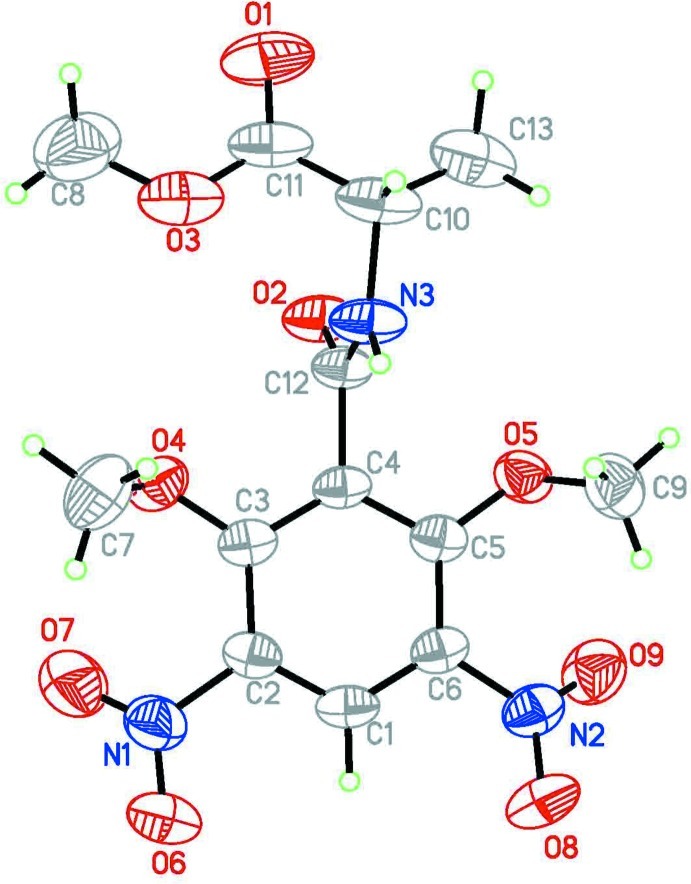
A view of the molecular strcuture of the title compound; the displacement ellipsoids drawn at the 30% probability level.

**Fig. 2.**
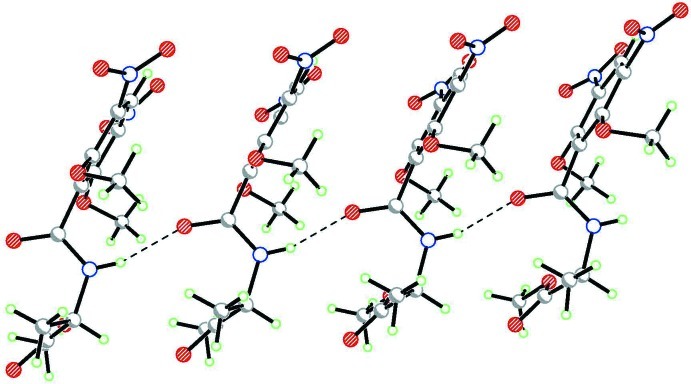
Part of the crystal structure showing C(3) chains along [100] direction. The hydrogen bonds are shown as dashed lines.

## supplementary crystallographic information

### Comment

Amides and Imides widely exist in many biological activity compounds or could be used as prodrugs (Sykes *et al.*, 1999). We synthesized the title compound and shall examine its biological activity. In the title molecule, C_13_H_15_N_3_O_9_, the nitro groups are tilted with respect to the benzene mean plane by 22.8 (3) and 31.6 (3)°. The methoxy groups are cis conformation. In the crystal structure the molecules are linked by strong N—H···O (H···O 2.02 Å; N···O 2.850 (2) Å; N—H···O^i^ 162° symmetry code: (i) 1+x, y, z) hydrogen bonds into C(3) chains along [100] (Bernstein *et al.*, 1995).

### Experimental

To a solution of D-alanine methyl ester hydrochloride (0.7 g, 5 mmol) and triethylamine (0.5 ml) in dry methylene chloride (100 ml) was added 2,6-dimethoxy-3,5-dinitrobenzoyl chloride 1.4 g, 5 mmol in dry methylene chloride (50 ml) at 0°C. The mixture was allowed to warm to room temperature for 1 h. After concentration the residue was subjected to chromatography (petroleum ether/ ethyl acetate, 3:1) to provide the product as a yellow crystal (1.3 g, 74.5%).

### Refinement

The C-bound H-atoms were included in calculated positions and treated as riding atoms: C—H = 0.93, 0.96 and 0.98 Å for CH(aromatic), CH_3_ and CH(methine) H-atoms, respectively, and N—H = 0.86 Å, with *U*_iso_(H)= k τimes *U*_eq_(parent C-atom, N), where k = 1.5 for CH_3_ H-atoms and k = 1.2 for all other H-atoms. Friedel pairs were merged and that absolute structure was determined relative to the known chiral centers.

### Crystal data

**Table:**

C_13_H_15_N_3_O_9_	*D*_x_ = 1.451 Mg m^−^^3^
*M**_r_* = 357.28	Mo *K*α radiation, λ = 0.71073 Å
Orthorhombic, *P*2_1_2_1_2_1_	Cell parameters from 9565 reflections
*a* = 4.6933 (10) Å	θ = 1.0–27.5°
*b* = 17.501 (3) Å	µ = 0.13 mm^−^^1^
*c* = 19.917 (4) Å	*T* = 296 K
*V* = 1635.9 (6) Å^3^	Block, yellow
*Z* = 4	0.31 × 0.30 × 0.09 mm
*F*(000) = 744	

### Data collection

**Table:**

Bruker APEXII CCD area-detector diffractometer	3683 independent reflections
Radiation source: fine-focus sealed tube	2824 reflections with *I* > 2σ(*I*)
Graphite monochromator	*R*_int_ = 0.025
φ and ω scans	θ_max_ = 27.5°, θ_min_ = 2.3°
Absorption correction: multi-scan (*SADABS*; Sheldrick, 1996)	*h* = −5→6
*T*_min_ = 0.962, *T*_max_ = 0.989	*k* = −22→15
9526 measured reflections	*l* = −25→25

### Refinement

**Table:**

Refinement on *F*^2^	Primary atom site location: structure-invariant direct methods
Least-squares matrix: full	Secondary atom site location: difference Fourier map
*R*[*F*^2^ > 2σ(*F*^2^)] = 0.045	Hydrogen site location: inferred from neighbouring sites
*wR*(*F*^2^) = 0.153	H-atom parameters constrained
*S* = 1.03	*w* = 1/[σ^2^(*F*_o_^2^) + (0.1*P*)^2^] where *P* = (*F*_o_^2^ + 2*F*_c_^2^)/3
3683 reflections	(Δ/σ)_max_ = 0.002
226 parameters	Δρ_max_ = 0.22 e Å^−^^3^
0 restraints	Δρ_min_ = −0.17 e Å^−^^3^

### Special details

**Table:**

Geometry. All e.s.d.'s (except the e.s.d. in the dihedral angle between two l.s. planes) are estimated using the full covariance matrix. The cell e.s.d.'s are taken into account individually in the estimation of e.s.d.'s in distances, angles and torsion angles; correlations between e.s.d.'s in cell parameters are only used when they are defined by crystal symmetry. An approximate (isotropic) treatment of cell e.s.d.'s is used for estimating e.s.d.'s involving l.s. planes.
Refinement. Refinement of *F*^2^ against ALL reflections. The weighted *R*-factor *wR* and goodness of fit *S* are based on *F*^2^, conventional *R*-factors *R* are based on *F*, with *F* set to zero for negative *F*^2^. The threshold expression of *F*^2^ > σ(*F*^2^) is used only for calculating *R*-factors(gt) *etc*. and is not relevant to the choice of reflections for refinement. *R*-factors based on *F*^2^ are statistically about twice as large as those based on *F*, and *R*- factors based on ALL data will be even larger.

### Fractional atomic coordinates and isotropic or equivalent isotropic displacement parameters (Å^2^)

**Table:**

	*x*	*y*	*z*	*U*_iso_*/*U*_eq_	
C1	0.7640 (5)	1.00166 (13)	0.58894 (9)	0.0458 (5)	
H1A	0.8771	1.0043	0.6272	0.055*	
C2	0.6630 (5)	1.06770 (14)	0.56054 (9)	0.0443 (5)	
C3	0.4986 (5)	1.06566 (13)	0.50149 (9)	0.0415 (5)	
C4	0.4388 (4)	0.99479 (13)	0.47345 (9)	0.0376 (4)	
C5	0.5350 (5)	0.92644 (13)	0.50238 (10)	0.0412 (5)	
C6	0.6998 (5)	0.93146 (13)	0.56147 (9)	0.0424 (5)	
C7	0.6194 (8)	1.17734 (18)	0.44049 (16)	0.0750 (9)	
H7A	0.5299	1.2192	0.4176	0.113*	
H7B	0.7360	1.1493	0.4095	0.113*	
H7C	0.7360	1.1967	0.4763	0.113*	
C8	0.0896 (12)	1.1761 (2)	0.26985 (16)	0.1048 (14)	
H8A	0.1719	1.2205	0.2905	0.157*	
H8B	−0.1055	1.1712	0.2836	0.157*	
H8C	0.0984	1.1813	0.2219	0.157*	
C9	0.6995 (7)	0.81397 (17)	0.44691 (14)	0.0634 (7)	
H9A	0.6247	0.7695	0.4248	0.095*	
H9B	0.8128	0.7985	0.4847	0.095*	
H9C	0.8154	0.8424	0.4160	0.095*	
C10	0.3449 (5)	0.97587 (18)	0.28526 (10)	0.0609 (7)	
H10A	0.5105	0.9771	0.2553	0.073*	
C11	0.1574 (6)	1.04345 (19)	0.26521 (11)	0.0605 (7)	
C12	0.2859 (4)	0.99044 (13)	0.40660 (10)	0.0414 (5)	
C13	0.2023 (7)	0.8995 (2)	0.27420 (14)	0.0836 (10)	
H13A	0.3295	0.8592	0.2875	0.125*	
H13B	0.1556	0.8938	0.2275	0.125*	
H13C	0.0314	0.8968	0.3006	0.125*	
N1	0.7169 (6)	1.13899 (13)	0.59792 (10)	0.0603 (6)	
N2	0.7960 (5)	0.86403 (13)	0.59826 (9)	0.0540 (5)	
N3	0.4560 (4)	0.98513 (13)	0.35366 (8)	0.0513 (5)	
H3A	0.6373	0.9872	0.3596	0.062*	
O1	−0.0446 (5)	1.03749 (15)	0.22813 (10)	0.0861 (7)	
O2	0.0251 (3)	0.99289 (11)	0.40308 (7)	0.0561 (5)	
O3	0.2451 (5)	1.10947 (14)	0.29000 (8)	0.0787 (6)	
O4	0.4046 (4)	1.12770 (10)	0.46763 (8)	0.0550 (5)	
O5	0.4680 (4)	0.86110 (9)	0.46968 (8)	0.0529 (4)	
O6	0.9142 (7)	1.13948 (14)	0.63661 (12)	0.1064 (10)	
O7	0.5635 (7)	1.19271 (14)	0.58904 (12)	0.0949 (8)	
O8	1.0181 (5)	0.87020 (14)	0.63012 (10)	0.0811 (7)	
O9	0.6518 (5)	0.80595 (12)	0.59661 (10)	0.0734 (6)	

### Atomic displacement parameters (Å^2^)

**Table:**

	*U*^11^	*U*^22^	*U*^33^	*U*^12^	*U*^13^	*U*^23^
C1	0.0423 (11)	0.0649 (13)	0.0303 (8)	−0.0062 (11)	−0.0056 (8)	0.0043 (10)
C2	0.0437 (11)	0.0547 (13)	0.0345 (9)	−0.0068 (10)	−0.0009 (9)	−0.0023 (9)
C3	0.0314 (10)	0.0576 (13)	0.0354 (9)	−0.0007 (10)	0.0013 (9)	0.0037 (9)
C4	0.0228 (8)	0.0588 (12)	0.0310 (8)	0.0009 (9)	0.0004 (7)	0.0008 (9)
C5	0.0260 (9)	0.0566 (13)	0.0410 (10)	−0.0022 (9)	0.0015 (9)	−0.0031 (9)
C6	0.0336 (11)	0.0581 (13)	0.0354 (9)	0.0022 (10)	0.0008 (9)	0.0048 (9)
C7	0.078 (2)	0.0735 (19)	0.0740 (17)	0.0061 (16)	0.0089 (16)	0.0259 (14)
C8	0.129 (4)	0.123 (3)	0.0616 (17)	0.032 (3)	−0.001 (2)	0.0052 (19)
C9	0.0620 (18)	0.0638 (16)	0.0644 (14)	0.0038 (13)	0.0064 (14)	−0.0134 (12)
C10	0.0324 (11)	0.118 (2)	0.0326 (9)	−0.0047 (13)	0.0007 (9)	−0.0132 (12)
C11	0.0410 (12)	0.110 (2)	0.0305 (9)	−0.0086 (14)	−0.0010 (10)	0.0026 (12)
C12	0.0239 (10)	0.0637 (14)	0.0365 (9)	−0.0017 (9)	−0.0035 (7)	−0.0036 (10)
C13	0.068 (2)	0.125 (3)	0.0582 (15)	−0.001 (2)	−0.0094 (16)	−0.0271 (17)
N1	0.0736 (16)	0.0621 (14)	0.0453 (10)	−0.0049 (12)	−0.0063 (12)	−0.0047 (10)
N2	0.0506 (12)	0.0682 (14)	0.0431 (10)	0.0058 (11)	−0.0007 (10)	0.0072 (9)
N3	0.0228 (8)	0.0949 (15)	0.0361 (8)	−0.0030 (9)	−0.0030 (7)	−0.0052 (9)
O1	0.0594 (12)	0.1340 (19)	0.0649 (11)	−0.0156 (13)	−0.0300 (11)	0.0121 (12)
O2	0.0222 (7)	0.1020 (13)	0.0441 (7)	−0.0014 (8)	−0.0028 (6)	−0.0035 (9)
O3	0.0758 (14)	0.1116 (17)	0.0487 (9)	0.0014 (14)	−0.0148 (10)	−0.0017 (10)
O4	0.0494 (10)	0.0585 (10)	0.0571 (9)	0.0025 (7)	−0.0093 (8)	0.0101 (8)
O5	0.0432 (9)	0.0580 (9)	0.0576 (9)	−0.0037 (8)	−0.0076 (8)	−0.0111 (7)
O6	0.139 (3)	0.0824 (15)	0.0981 (15)	−0.0124 (16)	−0.0724 (18)	−0.0102 (12)
O7	0.110 (2)	0.0825 (15)	0.0920 (14)	0.0233 (15)	−0.0242 (16)	−0.0324 (12)
O8	0.0694 (14)	0.0956 (15)	0.0782 (13)	0.0171 (12)	−0.0322 (12)	0.0102 (11)
O9	0.0795 (14)	0.0658 (12)	0.0747 (12)	−0.0045 (11)	−0.0042 (11)	0.0178 (10)

### Geometric parameters (Å, º)

**Table:**

C1—C2	1.371 (3)	C9—O5	1.438 (3)
C1—C6	1.378 (3)	C9—H9A	0.9600
C1—H1A	0.9300	C9—H9B	0.9600
C2—C3	1.407 (3)	C9—H9C	0.9600
C2—N1	1.475 (3)	C10—N3	1.468 (3)
C3—O4	1.352 (3)	C10—C13	1.512 (5)
C3—C4	1.389 (3)	C10—C11	1.527 (4)
C4—C5	1.402 (3)	C10—H10A	0.9800
C4—C12	1.514 (3)	C11—O1	1.207 (3)
C5—O5	1.353 (3)	C11—O3	1.322 (4)
C5—C6	1.411 (3)	C12—O2	1.227 (2)
C6—N2	1.461 (3)	C12—N3	1.326 (3)
C7—O4	1.436 (4)	C13—H13A	0.9600
C7—H7A	0.9600	C13—H13B	0.9600
C7—H7B	0.9600	C13—H13C	0.9600
C7—H7C	0.9600	N1—O7	1.197 (3)
C8—O3	1.433 (4)	N1—O6	1.205 (3)
C8—H8A	0.9600	N2—O9	1.222 (3)
C8—H8B	0.9600	N2—O8	1.225 (3)
C8—H8C	0.9600	N3—H3A	0.8600
			
C2—C1—C6	120.80 (18)	O5—C9—H9C	109.5
C2—C1—H1A	119.6	H9A—C9—H9C	109.5
C6—C1—H1A	119.6	H9B—C9—H9C	109.5
C1—C2—C3	120.86 (19)	N3—C10—C13	113.0 (2)
C1—C2—N1	116.46 (19)	N3—C10—C11	111.2 (2)
C3—C2—N1	122.5 (2)	C13—C10—C11	113.1 (2)
O4—C3—C4	116.79 (17)	N3—C10—H10A	106.3
O4—C3—C2	125.1 (2)	C13—C10—H10A	106.3
C4—C3—C2	118.00 (18)	C11—C10—H10A	106.3
C3—C4—C5	122.10 (17)	O1—C11—O3	123.3 (3)
C3—C4—C12	119.61 (18)	O1—C11—C10	123.1 (3)
C5—C4—C12	118.10 (19)	O3—C11—C10	113.6 (2)
O5—C5—C4	116.62 (18)	O2—C12—N3	123.90 (18)
O5—C5—C6	125.6 (2)	O2—C12—C4	121.40 (18)
C4—C5—C6	117.79 (19)	N3—C12—C4	114.68 (16)
C1—C6—C5	120.4 (2)	C10—C13—H13A	109.5
C1—C6—N2	116.97 (18)	C10—C13—H13B	109.5
C5—C6—N2	122.5 (2)	H13A—C13—H13B	109.5
O4—C7—H7A	109.5	C10—C13—H13C	109.5
O4—C7—H7B	109.5	H13A—C13—H13C	109.5
H7A—C7—H7B	109.5	H13B—C13—H13C	109.5
O4—C7—H7C	109.5	O7—N1—O6	123.4 (2)
H7A—C7—H7C	109.5	O7—N1—C2	119.1 (2)
H7B—C7—H7C	109.5	O6—N1—C2	117.4 (2)
O3—C8—H8A	109.5	O9—N2—O8	124.0 (2)
O3—C8—H8B	109.5	O9—N2—C6	119.2 (2)
H8A—C8—H8B	109.5	O8—N2—C6	116.8 (2)
O3—C8—H8C	109.5	C12—N3—C10	122.15 (17)
H8A—C8—H8C	109.5	C12—N3—H3A	118.9
H8B—C8—H8C	109.5	C10—N3—H3A	118.9
O5—C9—H9A	109.5	C11—O3—C8	116.6 (3)
O5—C9—H9B	109.5	C3—O4—C7	116.4 (2)
H9A—C9—H9B	109.5	C5—O5—C9	117.5 (2)

### Hydrogen-bond geometry (Å, º)

**Table:**

*D*—H···*A*	*D*—H	H···*A*	*D*···*A*	*D*—H···*A*
N3—H3*A*···O2^i^	0.86	2.02	2.850 (2)	162

Symmetry code: (i) *x*+1, *y*, *z*.
